# “We’re teetering on unsteady ground” parents’ experiences of accessing 24/7 paediatric end-of-life care: a qualitative study

**DOI:** 10.1186/s12904-025-01927-8

**Published:** 2025-11-12

**Authors:** Laura Barrett, Lorna Fraser, Lucy Ziegler, Stuart Jarvis, Susan Picton, Julia Hackett

**Affiliations:** 1 Paediatric Palliative Care Research Group, Department of Health Science, University of York, York, UK; 2 Cicely Saunders Institute, Dept of Women’s and Children’s Health, King’s College London, London, UK; 3 Academic Unit of Palliative Care, University of Leeds, Clarendon Way, Leeds, UK; 4 Leeds Teaching Hospitals NHS Trust, Beckett Street, Leeds, UK

**Keywords:** Paediatric end of life care, Paediatric care services, Palliative care, Qualitative research, Guidelines, Care pathways

## Abstract

**Background:**

Providing high quality around-the-clock care, is key to supporting families in their preferred place of care. Changing symptoms and parents’ distress cannot wait for ‘opening hours’. Yet in the UK, 24/7 children’s end-of-life care remains a significant postcode lottery. To inform equitable service development this study explored parents’ experiences accessing 24/7 paediatric palliative care, their expectations and needs.

**Methods:**

Qualitative study using in-depth interviews, analysed using thematic analysis. Parents in one region of England, were eligible if their child had a life-limiting condition and end-of-life care was planned, or if they were bereaved parents whose child had died within the previous 3–36 months.

**Results:**

Twenty-six parents were interviewed, 13 currently caring for their child and 13 bereaved parents. Two themes were developed: “Scaffolded for uncertainty and crisis” and “Falling through the service gaps”. Most parents want seamless 24/7 end-of-life care for their child at home and to avoid hospital admissions. Despite being desperate to be home and feeling unsafe in hospital, service gaps mean, for some families, there is no option other than their child dying as an inpatient.

**Conclusion:**

The study found marked inequity in parent’s experiences. Parents are confident when supported by a trusted 24/7 team with experience delivering palliative care, that provides phone support, face-to-face nursing and access to specialist advice. Hospital staff need improved training and consistent support from specialist palliative care teams. Further research with professionals is needed to understand the local and regional barriers that are preventing this support being available to all families.

**Supplementary Information:**

The online version contains supplementary material available at 10.1186/s12904-025-01927-8.

## Key statements

### What is already known on this topic?

- Providing good quality care and support, around the clock, across a range of services, is key to supporting children and their families’ in their preferred place of care.
- Families with a child at end of life are living in a state of precarity, and the distress of uncontrolled symptoms does not wait for ‘opening hours’, making it difficult for families to know what to do if something goes wrong out-of-hours and their usual health care services are not available.
- While parent preferences for setting of end-of-life care for their child are very personal, most studies report a substantial proportion of families would prefer their child to receive end-of-life care and die at home.

### What this paper adds

- Families supported by a 24/7 seamless service felt confident, assured and empowered to be responsible for end-of-life care for their child, while importantly maintaining some sense of ‘normal’ family life.
- Families not supported by a 24/7 seamless service felt abandoned by their daytime support, scared and alone.
- Taking their child to hospital was viewed as a last resort; lack of confidence in professionals to deliver safe, high-quality end-of-life care, the burden of caring for their child in an unfamiliar setting, and risk of exposure to infections meant they felt unsafe.

### How this study might affect research, practice or policy

- Commissioned high quality end-of-life care needs to be a seamless 24/7 service, that consists of phone support from a trusted professional, face-to-face nursing and access to specialist advice.
- To ensure safe, high-quality in-patient end-of-life care, ward staff need improved professional training, particularly around symptom control, and consistent support from specialist palliative care teams.
- Further in-depth research with service providers and healthcare professionals is needed to understand the local and regional barriers, and to inform the provision of an equitable 24/7 seamless support service.

## Background

Children with life-limiting conditions are living longer with increasingly complex symptoms [1]. All children and their families should have access to individualised care including choice of setting for end-of-life care and place of death [2–4]. Providing high quality care day and night, is key to supporting families’ choice[5, 6], as changing symptoms, pain or parents’ distress cannot wait for ‘opening hours’[7].

International and national standards including the European Association of Palliative Care’s Charter for Paediatric Palliative Care[8], the UK’s NHS Ambitions for Palliative and End-of-life Care [9] and National Institute for Health and Care Excellence’s quality standards [6] all recognise that families caring for their child at home should have access to around the clock support. Yet in the UK, there remains a significant ‘postcode lottery’ of how 24/7 services are planned, funded and provided [10]. The recently released palliative care research priorities from Marie Curie highlight the need to further understand the best way to provide end-of-life care around the clock [11]. And while survey data can highlight the issues[10], to develop equitable access to services that meet families’ needs, it is important to fully understand parents’ perspectives of accessing and receiving 24/7 end-of-life care for their child [12, 13]. 

This paper is part of a wider study which aims to support the planning and provision of 24/7 paediatric palliative care, by exploring parent and professional experiences and needs, the patterns of care use at end-of-life, and to identify the key components of a round-the-clock service intervention in one English NHS region. This will ensure new services developed are grounded in evidence, reflect the current service landscape and work in everyday practice, so that children and their families will have access to specialist medical advice and skilled nursing support where, when and how they need it.

The aim of the workstream described in this paper, explored families’ perspectives on the accessing 24/7 paediatric palliative care across the region, and their expectations and needs of a new service. Results from other workstreams of the study, including the perspectives of healthcare professionals, are reported elsewhere [14]. 

## Methods

This is a qualitative study using in-depth interviews and thematic analysis. An interpretivist approach was taken to understand the subjective meaning of parents’ lived experiences of 24/7 end-of life care within context, whilst recognising the importance of researcher influence in such interpretations [15, 16]. The study is reported in accordance with Consolidated Criteria for Reporting Qualitative Research (COREQ) guidelines [17]. 

### Public and patient involvement (PPI)

A parent advisory panel of five parents with diverse experiences advised throughout the study, from application to dissemination. The panel included both bereaved parents who had accessed end-of-life care and those with a child receiving palliative care. One parent was a member of the Study Steering Committee. Input included piloting the interview guide, widening the recruitment strategy, and advice on sensitively discussing preferences for place of death with parents whose child was receiving care.

### Ethics

Ethical approval s were obtained from the Health Research Authority and Health and Care Research Wales (21/10/2022, 317352). Research governance approval was also secured at each participating NHS trust, hospice and recruitment site in accordance with their local procedures.

### Population

Parents within a region of England were eligible if their child had a life-limiting condition and end-of-life care had been discussed or if they were bereaved parents whose child had died between 3and 36 months previously. Inclusion criteria for bereaved parents were informed by recent literature[18–20], team experience, and discussion with parent advisors.

### Sampling and recruitment

To ensure a diverse sample and to address the parent advisory panel’s concerns about practitioner gatekeeping, a multi-pronged approach for recruitment was used. Parents were recruited via three tertiary NHS sites, nine regional children’s hospices, parent-facing organisations and social media platforms. Participants were purposively sampled according to characteristics of their child including diagnosis, age and geographical area.

Recruitment sites identified eligible parents, and those interested were given a brief information sheet and consent-to-contact form. This form was returned to the study team who sent parents an invitation and detailed information. The team followed up with a call to check eligibility, build rapport, give participants a chance to ask questions and to arrange the interview. Interested parents who read about the study via social media, contacted the study team directly and were sent detailed information and followed up as above.

### Data collection

In-depth interviews explored parents’ experiences of accessing 24/7 end-of-life care for their child. Parents could choose a face-to-face, telephone or video-call interview. Informed consent was taken before each interview and monitored throughout. Where both parents wished to participate, individual or joint interviews were offered. Interviews were undertaken by two authors (LB and JH; both females, applied health researchers, and previously unknown to participants). See supplementary material for topic guide. Interviews were recorded and transcribed. To limit participant burden transcriptions were not returned to parents for checking. Researchers were experienced in conducting sensitive interviews and the possibility of distress was explicitly discussed with all participants. Researchers reflected and debriefed after each interview, iteratively revising topic guides. These conversations were also helpful for analysis and sense checking interpretation. Protocols were in place to signpost both participants and researchers for further support if required.

### Data analysis

The analysis followed Braun and Clarke’s six-stage approach to thematic analysis, which is well suited to an interpretivist stance [15]. This approach treats themes as patterns of shared meaning that are actively generated by researchers through iterative coding and interpretation, rather than as topics waiting to be discovered [15, 21]. For data familiarisation, LB listened to all audio-recordings, then read interview transcripts to gain a sense of parents’ experiences, with notes taken on key concepts. LB then used both inductive coding, staying close to participants words and deductive coding based on the interview guide. Initial codes were then grouped into descriptive categories to explore potential relationships, and divergent cases were sought. LB and JH iteratively developed analytical themes by seeking patterns of shared meaning in coded data and descriptive categories [15]. Themes were collaboratively named, defined and refined through discussion with the parent advisory panel and other research team members. NVivo 14 was used for managing data [22]. 

### Reflexivity statement

Researchers’ existing knowledge and experience was acknowledged as necessarily shaping the research process including data collection and analysis. The wider team and external stakeholders including the parent advisory panel, provided additional perspectives and helped draw out and interpret parent experiences, creating a more balanced and gender-diverse team. The team critically reflected throughout the process including around methods chosen, the topic guide, and the analysis and interpretation of findings.

## Results

### Sample

Twenty-six parents of 26 children were interviewed, of which 13 were bereaved parents, and 13 were caring for a child with a life-limiting condition. See Table 1 for an overview of sample characteristics. Two interviews were with couples and one interview covered three children. Interviews took place April 2023 to April 2024 and mean interview length was 68 min (range 23–121 min).

**Table 1:** Overview of sample characteristics

	Bereaved parents	Parents of children receiving care
**Characteristic of children** **(** ***n*** **= 26)**		
Sex of child		
Male	6	9
Female	6	5
Age of child (at time of interview or at time of death)		
0–5	4	4
6–15	7	7
15–19	1	3
Diagnosis Group		
Cancer	5	0
Metabolic	3	4
Neurological	2	4
Perinatal	0	1
Congenital	2	5
Siblings		
Yes	11	13
No	1	1
Place of death		
Hospital	3	n/a
Home	9	n/a
**Characteristics of parents** **(** ***n*** **=26)**		
Mothers	11	10
Father	2	3
Ethnicity		
White British	10	10
Other	3	3

All but one of the families had received end-of-life care from either an NHS based specialist palliative care team (*N* = 10), a hospice team (*N* = 14) or a paediatric oncology team (*N* = 5). Several families were supported by both an NHS palliative care team and a hospice team. None of the families of a child with cancer were supported by a hospice. A specialist children and young people palliative care team is defined as including a doctor with specialty training (a consultant) in paediatric palliative medicine [23]. 

### Themes

**Fig. 1:**
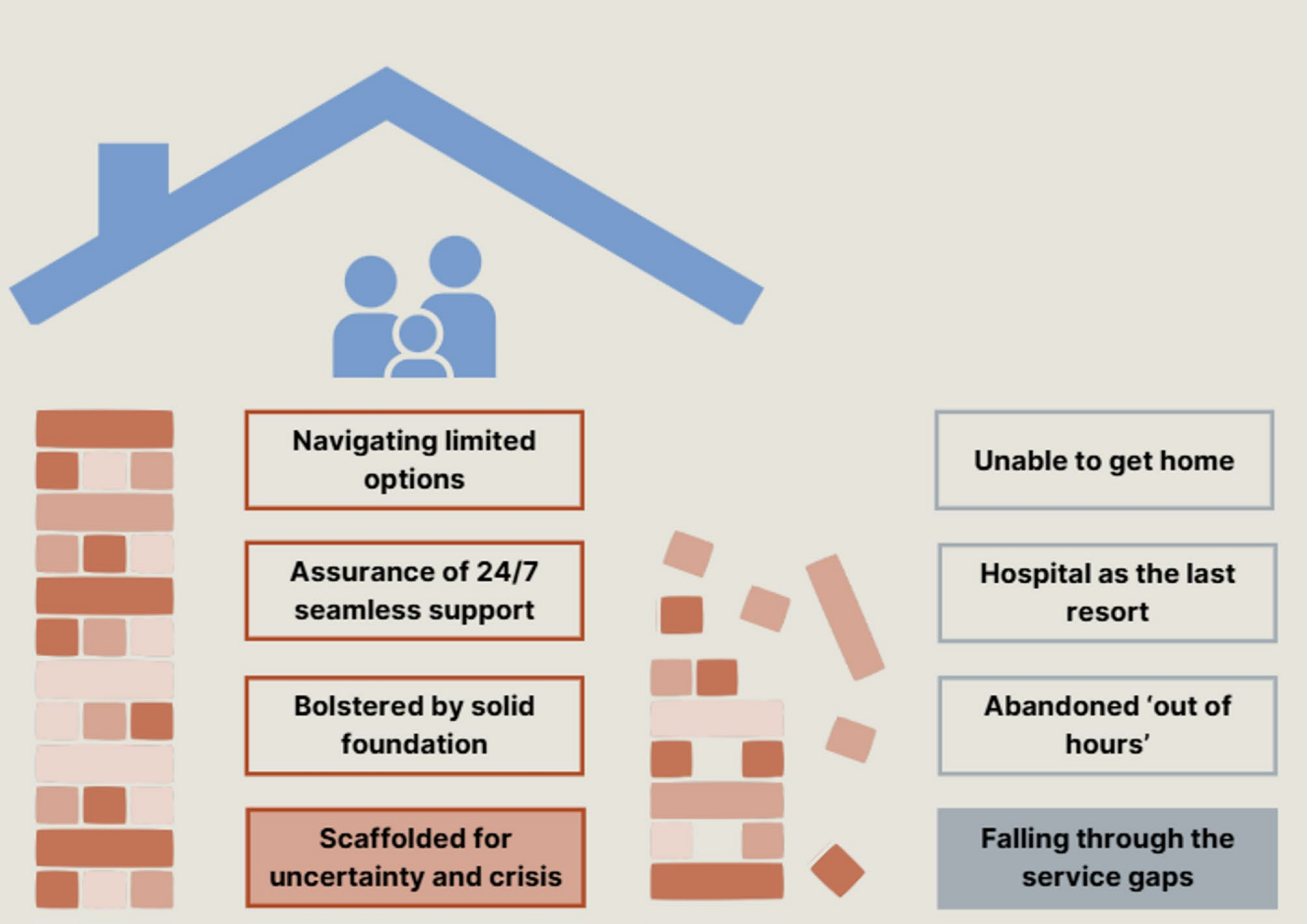
Themes and subthemes

Two themes with six subthemes (see Fig. 1; Table 2) were developed from parents’ experiences of accessing and receiving 24/7 end-of-life care for their child: “Scaffolded for uncertainty and crisis” and “Falling through the service gaps”.

**Table 2:** Subthemes and their definitions

Theme	Subtheme	Description	
Scaffolded for uncertainty and crisis			Parents’ experiences receiving and navigating the 24/7 support available to them in precarious times
	Bolstered by sold foundations	Having the support of a trusted and experienced team gave parents confidence	
	Assurance of 24/7 seamless support	Parents valued having access to the same known core team whenever needed	
	Navigating limited support option	In an emergency, some parents had to navigate the available support options	
Falling through the service gaps		Impact on families who experienced service gaps	
	Abandoned ‘out of hours’	Parents’ felt vulnerable when services were closed	
	Hospital as the last resort	Parents’ experiences of feeling unsafe and unsupported in hospital	
	Unable to get home	Families’ experiences of being stuck in hospital	

### Scaffolded for uncertainty and crisis

Parents had a common awareness that they faced uncertainty, “*we live on a ticking timebomb*” (04–19) and their child’s health and therefore the family’s day to day functioning was precarious. Families wanted a solid foundation of trusted support to enable them to be their child’s main caregiver, while recognising there were times that they needed the security of a responsive service, including at weekends and evenings. However not all families had access to same level of support around the clock, and many had to navigate the available provision.

#### Bolstered by solid foundations

Depending on where they lived, families were supported by different types of services. All but one family in the study were under the care of a team with experience delivering palliative care: either an NHS based specialist palliative care team, a hospice care team or an oncology care team. These teams were usually the main point of contact for families who recognised their specialist skills and knowledge, particularly for symptom and pain management.

*[Child’s] life changed when [hospice] got involved. She got medication that [local hospital] were too scared to give her. She got treatments that [local hospital] wouldn’t refer – like her life got better once she became under palliative care. If you say that*,* that sounds like weird*,* but it did. Her quality of life improved*,* and ours as a family*,* ‘cos we were so isolated before. (08–34)*

Families formed close and trusted relationships with the teams, who then were able to understand personal contexts, preferences, cultures and beliefs. Oncology teams had often been involved since a child’s diagnosis, and parents valued this continuity.

*I think having a set keyworker who knew the ins and outs of [child’s] treatment and where our routines were and what appointments we’re having and where she went to school*,* I think that were amazing. (01–04)*

This trust enabled families and professionals to have difficult conversations, including discussing with parents who wanted their child to die at home, arrangements around the child’s death. This gave them a sense of control.

*So*,* [Specialist palliative care nurse] knew my wishes*,* that I wanted to keep [Son] here. We spoke about it right at the beginning when we wrote the advance care plan. [.] She knew how desperate I was to keep him with us*,* and they made that happen. (03–22)*

The teams’ palliative care skills and experience enabled them to support parents with planning despite the unpredictability of end of life phase. Parents described feeling reassured by having a step-by-step plan that they knew other professionals also had access to. Although a few parents did still worry if plans had been disseminated to the right people and if they would be followed in an emergency.

*But she also wrote up a symptom control plan so that’s been sent to my GP*,* our local A&E*,* our local assessment unit*,* [.] they’ve literally got step by step instructions on what to do to stabilise him*,* on what medicines he can give him. Cos that was a massive worry of mine. So the fact that she’s done that symptom control plan*,* we can now use that and say this is what he can have and we’ve got it in writing. It made the world of difference. (01–02)*

Parents also valued the team’s holistic approach, and this gave parents the confidence to be at home, so they could bring a semblance of normality and to sustain their child’s, and any sibling’s, broader quality of life.

*So I took over all of [daughter’s] care. Everything came home. […] I would just do it all at home*,* just to keep us all happy really and*,* you know*,* a bit of normal life going as much as it could. (11–15)*

#### Assurance of 24/7 seamless support

In parts of the region, parents experienced a continuous seamless 24/7 service. These families were supported by either a larger hospice care team or an oncology outreach team who were available day and night. Parents valued knowing the same professionals were on standby and felt it was ok to ring any time, even in the middle of the night, rather than waiting until morning. They felt added confidence that the team on the end of the phone knew their child and the specifics of their illness. Parents who had this level of support felt assured and were universally appreciative.

*It was more someone who knew [child] inside out*,* who knew us as a family inside out and if I needed her at two o’clock in the morning*,* she’d be there (11–15)*.

The service model of how support was delivered out-of-hours varied considerably across the region, with one hospice providing a doctor-led 24-hour phone support with home visits if required, whereas another hospice had a nurse-led support service while an oncology outreach team had a rota of on-call nurses who regularly attended to families’ homes if needed.

*[Oncology nurse] said*,* “Just ring us 24/7*,* you know*,* you can ring me any time…*,*” and we were given on*,* on call rota again (01–04)*.

Often families just wanted to speak to a familiar professional for reassurance, maybe to double check a drug dosage or confirm a course of action. Easy access and clear signposting of how to reach a service out-of-hours added to parents’ sense of feeling supported.

*The hospice is 24/7 and that is the beauty of it. We can send them a message or pick that phone up at any time and there’s somebody. Even if it’s only a bit of a reassurance over something sometimes. It*,* it means the world*,* don’t it? (05–16)*

Other times families needed someone to come to the house during the night, this could be for more practical support such as changing batteries in machines or sourcing prescriptions, or they could need clinical advice.

*She started with some blisters around the top of her legs*,* and we didn’t know if it were the catheter had leaked or whatever it were. Anyway*,* I rung the [Oncology team] rota and she came straight out and it were shingles. (01–04)*

Families described how being able to access advice from a specialist doctor was particularly important, although often just being able to consult was sufficient, they did not automatically need in-person support.

*It’s clinical support when you need it that is the big thing. They don’t need to come out to you…all I need to do is just FaceTime and say*,* have a look at this rash*,* this is an interesting one. If they then think it’s serious enough to actually come and have a look at themselves*,* then that’s fine. But it’s just having someone that’s confident really. (03–30)*

As a child moved towards their end of life, parents’ felt empowered to continue to care for their child at home, by proactive and dependable support, with teams checking in regularly and ‘stepping up’ support if required.

*And on the days where she didn’t visit*,* she’d text me in the morning to say*,* do I want her to come*,* do I want her to look over [child]. So that happened for about a week which was really reassuring and really supportive. (03–22)*

Parents who felt confident and comfortable to be alone at home with their child as they died, did not need to worry about who to call when the time came. They were able to reach their care team at any time, who responded promptly, and arrived at the house to ensure necessary arrangements were made.

*Then at three o’clock in morning*,* when she actually passed we just rung [Oncology nurse] and she came out*,* I’m glad it were her. She came out really quickly*,* it probably be*,* 20 min*,* half an hour away*,* so*,* but she came out really quickly and she did all paperwork and whatever you need to do when you pass away. (01–04)*

Parents who experienced a fully supportive 24/7 seamless service throughout their child’s end-of-life, were clear they had no regrets about how their family were cared for and were grateful for the support they had received.

*As I say*,* I think we can look back and say that genuinely we didn’t have any regrets about the way anything was done*,* you know*,* it was a horrible situation that we wouldn’t have wished for but knowing that’s the situation we were in*,* we can’t really look back and say well that could have been better or that should have been different. (00–01)*

#### Navigating limited support

The scaffolding of services to support families around the clock was inconsistent across the region, and NHS based specialist palliative care teams were mostly small, with part-time staff, and only able to support families during office hours. Smaller hospices were also only able to provide daytime support. While some families had robust daytime support from children’s community nursing teams, few if any of these teams, were available in evening or weekends, and there were areas where there was no community nursing team at all.

Out of ‘office’ hours, if there was an emergency, families navigated accessing other services, who they were unfamiliar with or in some cases, never used before. In one area, the palliative care team handed some families to an overnight phone line not directly involved in a child’s routine care. There were often gaps in communication and parents did not feel this was a helpful or reliable support. They found it difficult when the staff on phone did not know their child and did not have the required expertise/specialist knowledge and as a result usually ended up advising the family to take the child to hospital.

*You can ring but they’re not as up to date on patients if they are not their patients. So the palliative team do sometimes leave them a sort of plan of action for when they aren’t in. [.] They don’t know [Daughter]*,* they can only go off by the book suggestions and like I said*,* [Daughter] is definitely not a by the book kind of girl. […] There’s not always a prescriber on shift*,* which is not helpful. (03–24)*

Other options for families included accessing out-of-hours GP, phoning 111, taking their child to hospital or calling an ambulance. Parents described the stress of facing a situation where they needed support but had to work out what was the best course of action, who was the most appropriate service and then understanding the consequences of their actions.

*There was no way I could ring a normal out-of-hours service and say “just to let you know*,* I’ve got a child on end of life and I’m struggling a bit here”. I was petrified at the idea that someone who didn’t know what they were doing would come*,* panic themselves and phone an ambulance and then she ended up dying in hospital (02–14)*.

Services covering large geographies meant families travelling long distances to access care. Families were also frustrated by administrative boundaries that meant they were unable to access the most local service, and by clear difference of provision within small areas.

*It was frustrating because we’re in the*,* we’re in the middle of two hospitals. [.] So [Tertiary Centre] they have community nurses that work longer hours*,* that come out when the NG tube’s done*,* whereas [local trust] if it comes out*,* they say they only work ‘til four o’clock. (02–35)*

### Falling through the service gaps

The inconsistency of 24/7 support at home for families across the region, meant some parents faced situations where they had no choice over how and where their child was cared for. This theme describes the impact on families who experienced service gaps.

#### Abandoned ‘out of hours’

Parents who were caring for their child at home but lived in areas where there was no access to out-of-hours support from a hospice or an NHS palliative care team, described the impact this had on their lives. These parents were all keenly aware of when their team was ‘open’, and this meant anticipating care needs and potential issues. This preparation work meant that parents expended precious time and energy, planning and making sure advice, supplies and prescriptions were all in place before they were left on their own to cope with whatever arose.

*It’s a little bit of a panic for us as well cos we’re like*,* oh God*,* we’ve gotta get it done before they go off for weekend or oh God*,* we gotta get it done before they go off at night. (03–24)*

Families’ needs did not change because it was a different time of day, and they faced trying to manage with difficult and frightening situations until their team were available to help.

*She got pneumonia on Sunday afternoon and we had no one*,* as you know*,* because it’s not working hours*,* it’s not 9:00 to 5:00*,* Monday to Friday*,* which weren’t ideal. […]. So*,* I messaged [NHS palliative care nurse] when she started getting poorly*,* hoping that we could keep her safe at home until Monday morning when they were all back at work. (03–24)*

Parents described feeling scared and abandoned when the professionals they trusted and relied on during the day were then not available out-of-hours.

*The community team were available like 9am till 6pm*,* and then they’d have an answerphone service. And weekends*,* it was I think till lunchtime*,* so… Before we had [hospice]*,* that were really scary*,* really scary. I think they’d go into work and have about ten voicemails from me*,* like*,* “Please ring me*,* please ring me*,*” you know. (08–34)*

They contrasted the feelings of confidence and being in control during the day, with the isolation of night-time.

*I think with anything*,* the support you need is on a nighttime. I think during the day everything else seems okay*,* you know? You’ve got daylight*,* you’ve got shops open*,* you’ve got the hospitals open. On a nighttime everything just feels like you’re alone and it’s just you and the poorly person. and on weekends you feel alone because there’s not that service there*,* erm*,* and I think that that’s hard. (02–35)*

Being a child’s primary caregiver took its toll on the physical and mental health of parents, pushing them to the limits “*it was just relentless*,* relentless all of the time*” (02–35) but without back up support there was no-one to take over. If they wanted to be at home with their child, they had no choice but undertake new medical responsibilities and this was distressing for both them and their child.

*You’ve got nobody there [.] and then in the end*,* [Dad] learned how to*,* how to pass the tubes himself and he’d done that and that killed him. It was distressing for him. […] it took away from him being a dad almost*,* if you know what I mean? It was really hard on him. (02–35)*

Parents whose child was receiving end-of-life care in hospital also described feeling abandoned out-of-hours. NHS based specialist palliative care teams’ presence in the hospital ward during the day to support parents and staff was welcomed. Parents appreciated the arrival of a familiar face who provided continuity of care, and they were reassured when these specialists advised other staff about care plans and symptom management. However, when the specialist teams were only available during working hours, parents were frustrated by the service gaps, and felt their child was not consistently getting the care required and in some cases was left in pain.

*I remember being in hospital when she was dying and like wishing the hours to go so*,* I could ring [specialist paediatric palliative care nurse] and say*,* “you need to come.” Like*,* “Please look on the system*,* this has happened*,*” or “She’s not had this medicine and we’ve been waiting this long*,*” (08–34)*.

#### Hospital as the last resort

Families usually had an option to contact a local children’s ward and had been given a letter of ‘open access’. This back up system worked well for some parents and was described as a comfort.

*We’d just pack everything for me and her and I would just head up to hospital*,* no matter what time - we had open access*,* which used to be brilliant*,* (02–14)*

For one child with cancer, the hospital oncology ward was familiar as they had often spent protracted time receiving previous treatments and so felt comfortable there.

*[Daughter’s] safe place*,* whenever she didn’t feel well*,* were to ask to go to [Tertiary hospital] (01–04).*

Ultimately most families who had choice around the place of end-of-life care for their child hoped to be at home for as long as possible, with a few planning to move into the hospice as their child died. Many families, particularly those with children who had a non-cancer diagnosis were not confident in the care they would receive in hospital, and nearly all parents spoke of trying to avoid going until it became an absolute necessity. The weight of the decision to take their child into hospital was intensified by a worry they could also be putting them at risk of infection and their health might deteriorate further.

*I mean*,* I’m not fully against taking her into hospital if she needs it*,* I’d just rather not because she tends to catch twelve more infections than what she went in with and that’s just not good when we’re teetering on unsteady ground with’ [daughter] (03–24).*

When parents realised they had no option but to take their child in, the process of accessing care was inconsistent: in some places children were admitted swiftly and directly, whilst others had to wait for long stretches in A&E or an admissions unit.

*So*,* erm*,* we*,* we have open access to the ward. Erm*,* but to get to the ward*,* we have to go through paediatric day unit or children’s day unit. We can’t go straight to the ward because obviously*,* they need time to get bed ready and whatever*,* erm*,* and that’s where the problem… that*,* that can cause problems because you can end up getting stuck in the*,* in the PDU or the A&E for hours and hours. (04–19)*

Once a child was admitted, families found themselves on wards where they perceived staff to be over stretched and under-resourced and where the required facilities and equipment was not available. Parents were at this point often already exhausted from the run up to an admission, but found they were still responsible for the bulk of a child’s care, in what were now, much more difficult circumstances.

*We are saving the NHS a fortune because we are keeping this child out of hospital numerous times a year*,* erm*,* so when we do end up in hospital with him*,* we’ve already*,* we’re already exhausted because we’ve already spent three or four days pretty much round the clock trying to prevent him from being*,* landing in hospital so by the time we reach it*,* we’re already a little bit tired*,* you know*,* and we don’t need to be doing your job*,* you know*,* so but inevitably we do (08–21)*.

*The hospital was horrific. I felt like we rented a room. We did all her care. (08–34)*

Parents felt the complexity of caring for a child at the end-of-life required a level of expertise and experience not always available on a general ward or in the local hospitals.

*They [the nurses] were absolutely petrified. And we had it said on more than one occasion that the nurses aren’t there to look after children like (child) when they’re dying. (08–34)*

This led to frustration from parents who had to continue to act as their child’s advocate, fighting for appropriate care, and in some cases educating staff about administering sufficient levels of pain relief.

*She wasn’t doing well at all [.] but the two nurses didn’t know what they were doing and [Dad] had to go out and hunt them down and say*,* “Have you looked at the care plan? Have you looked at what she’s allowed because she’s allowed this now and you’re not giving her it?” (02–35)*.

Parents continued to assess the risk of a child staying, and in some cases made the decision to leave as soon as was feasible.

*In the end*,* we’d had half an hour sleep in 72 h and we just thought I can’t*,* can’t do this anymore and she*,* she’s not safe here and we’ve got all the equipment we need at home (03–30)*.

#### Unable to get home

In some cases, as a child in hospital reached the end-of-life, and they needed intense medical support around the clock, the specialist services required to support a child and their parents to get home, were simply not available. Parents who had had previous experiences of adult family members dying at home, were surprised and shocked the same level of support was not in place for children.

*I was just told that that they just didn’t do it. I mean*,* palliative care nurses themselves would been finished at*,* I think their shift ended at six o’clock and wasn’t any provision for anything else. That was it. So there was no one*,* no nursing team*,* there’s no charities that done anything like that. […] it was just I wanted my little girl to be in my arms at home on the sofa with us*,* [Dad] could be watching a film*,* we could just have that time. I*,* I*,* I didn’t want anything else other than that*,* you know (02–35)*.

Ultimately the lack of 24/7 provision from either the NHS or a local hospice meant families were not able to be at home and had to remain in hospital while the child died. These bereaved parents were left with the knowledge their child had died in a place they had not wanted to be, and this remained with them.

*But I never wanted her to die in hospital. That wasn’t what we wanted*,* ‘cos she hated the hospital. [.] And because of how ill she was*,* the palliative team in [town x] advised us that we wouldn’t be able to care for her at home*,* the amount of medication that she was having*,* and the doses changing every day through the night. There’s nobody in [town x] to come and administer them medications at two in the morning. (08–34)*

## Discussion

### Main findings

The study found marked regional inequalities in parent’s experiences of accessing end-of-life care for their child. Most families wanted their child to be cared for at home for as long as possible. Parents felt bolstered by a trusted team with palliative care experience who support planning and empower parents to balance caregiving with parenting. Recognising the unpredictability of the end of life period for a child, parents expressed a need for a seamless 24/7 service on hand to provide phone advice, with the option of face-to-face nursing support and access to specialist advice from consultants if needed.

Access to palliative care depended on local services and a child’s diagnosis. Support varied from NHS specialist palliative care teams, hospice care teams, or children’s oncology teams. Some teams provided seamless care day and night, while others only offered ‘in-hours’ support, leaving families feeling scared and abandoned. For many parents, taking their child to hospital was often the only option, despite it not feeling safe.

### What this study adds

Previous research acknowledges the importance of 24/7 access to professionals with palliative care knowledge for home-based end-of-life care[4, 5, 13, 24], and this is reflected in UK and international quality standards and service specifications [6, 8, 9, 23]. However this research confirms regional disparities [10] and goes further to highlight the impacts on parents when this support is unavailable, resulting in inequities in experiences and outcomes.

Good paediatric palliative care is integrated, responsive and flexible, and should enable parents to care for, and also to parent their child at the end of life [4, 12, 25]. This study shows families supported by a seamless 24 h-a-day service, felt confident and empowered to deliver end-of-life care for their child, while maintaining some sense of ‘normal’ family life.

Families who did who did not have 24/7 access to their team felt a sense of abandonment by their daytime support which adds to social isolation [26]. Research shows while 24/7 telephone advice lines are increasingly used in palliative care[27], including for adults, there is little evidence of clinical and cost effectiveness [28]. When families were ‘handed over’ to an advice line, inefficiencies between in-hours and out-of-hours services meant key information was often not communicated. Parents did not find it helpful to contact professionals who did not know them or their child.

When at-home palliative care support was not available, taking the child to hospital was often the last resort. The burden of caring for their child in an unfamiliar setting, was confounded by risk of infections and lack of confidence in ward staff to deliver safe end-of-life care. Parents described the distress of seeing their child in pain, while waiting for staff to understand and administer pain relief. Research has shown health professionals’ lack of palliative care experience can be an underlying cause of patient safety incidents [29]. This study adds evidence that when hospital staff are supported by paediatric palliative care specialists, parents felt symptom management improved, which is in accordance with previous research [30, 31] but when this support was not available parents were left alone with a child in pain.

Findings reflect previous research on the importance to many parents of their child’s end-of-life care being delivered at home [24, 32]. NHS Specialist Palliative and End-of-life Care Services specifications are clear families should be supported in their preferred place of care and death[23], and crucial outcome of high quality paediatric palliative care is the alignment between preferred and actual place of death [32]. A key barrier identified in this study is a lack of 24/7 home support for children with significant care needs at the end of life. Gaps in services, meant for some families, despite being desperate to be at home, their child died in hospital.

### Strengths and limitations

A study strength is that it explores in-depth the perspectives of parents receiving a range of models of care and highlights the inequalities in their experiences. A multi-pronged recruitment strategy ensured the sample had a good representation of parents with different aged children, varied diagnoses and from across the region. Whilst 6 out of 26 parents self-identified as non-white, this is broadly reflective of the region’s demographic diversity, however further focused research would be needed to identify the specific needs of minority populations. Participants included both bereaved parents who described their experiences of support when their child died, and parents caring for a child where end-of-life care was being delivered or planned. However, the voices of parents of children with cancer currently receiving end-of-life care were not captured. Only about quarter of the research participants were fathers, making it difficult to differentiate between fathers’ and mothers’ experiences.

### Implications for policy/practice

This research focuses on the inequalities arising from regional variations in access to 24/7 paediatric end-of-life of care. Commissioners must consider the components parents expressed as key to effective high quality 24/7 service: continuous and seamless access to a trusted and experienced palliative care team including phone support, face-to-face nursing and specialist advice.

Hospital was not always seen as a place of safety for families with a child at the end of life. Improved health care professional training in palliative and end-of-life care particularly around symptom control, and consistent 24/7 input from specialist palliative care teams would improve the quality of inpatient care.

Despite the recognition of the importance of 24/7 end-of-life care in quality standards and service specifications, these fall short in implementation and delivery. Further in-depth research with service providers and healthcare professionals is needed to understand the local and regional barriers, and to inform the delivery of an equitable 24/7 seamless support service for children at the end of life, and their parents.

## Conclusion

The study found inequity in parent’s experiences. Parents need access to a trusted and seamless 24/7 palliative care team, providing phone support, face-to-face nursing and specialist advice when needed. This will enable families who prefer to be at home for their child’s end-of-life care, to stay there for as long as possible and avoid hospital admissions. Further work with service providers and healthcare professionals is required to tackle the local and regional barriers preventing this support being available to all families.

## Supplementary Information

Supplementary Material 1

## Data Availability

The dataset generated and analysed during the current study is not publicly available due to ethical considerations (to ensure data confidentiality and protect the anonymity of the research participants) but are available from the corresponding author on reasonable request.
