# Supplementary material for: “We’re teetering on unsteady ground” parents’ experiences of accessing 24/7 paediatric end-of-life care: a qualitative study
Source: BMC Palliat Care. 2025 Nov 12;24:285. doi: 10.1186/s12904-025-01927-8 (PMC12613421; doi:10.1186/s12904-025-01927-8)
Supplement: Supplementary file 1 — Supplementary Material 1 [file 12904_2025_1927_MOESM1_ESM.docx]

**
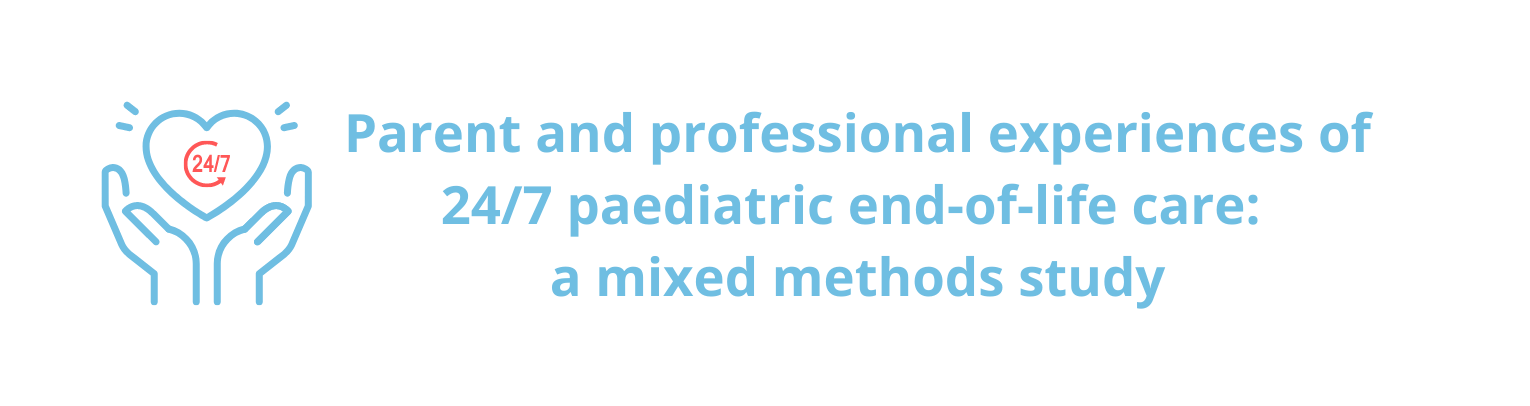
**

**Topic guide for semi-structured interview with bereaved Parents**

**Introducing the interview and consenting process**

**Finding out about the child and family**

**History of the child’s condition and general care use including out-of-hours**

**Planning and decision making**

**Care at the end-of-life**

**Out of hours care**

**Learning from what works well and less well**

**Expectations and needs of a new service**

**Close.**

**Topic guide for semi-structured interview with Parents – non-bereaved**

**Introducing the interview and consenting process**

**Finding out about the child and family**

**History of the child’s condition and general care use**

**Planning and decision making**

**Current access to 24/7care**

**Changing care needs and moving to end-of-life care**

**Learning from what works well and less well**

**Expectations and needs of a 24/7 service**

**Close**
